# Reduced Metabolic Cost of Locomotion in Svalbard Rock Ptarmigan (*Lagopus muta hyperborea*) during Winter

**DOI:** 10.1371/journal.pone.0015490

**Published:** 2010-11-15

**Authors:** John Lees, Robert Nudds, Karl-Arne Stokkan, Lars Folkow, Jonathan Codd

**Affiliations:** 1 Faculty of Life Sciences, University of Manchester, Manchester, United Kingdom; 2 Department of Arctic and Marine Biology, University of Tromsø, Tromsø, Norway; Pennsylvania State University, United States of America

## Abstract

The Svalbard rock ptarmigan, *Lagopus muta hyperborea* experiences extreme photoperiodic and climatic conditions on the Arctic archipelago of Svalbard. This species, however, is highly adapted to live in this harsh environment. One of the most striking adaptations found in these birds is the deposition, prior to onset of winter, of fat stores which may comprise up to 32% of body mass and are located primarily around the sternum and abdominal region. This fat, while crucial to the birds' survival, also presents a challenge in that the bird must maintain normal physiological function with this additional mass. In particular these stores are likely to constrain the respiratory system, as the sternum and pelvic region must be moved during ventilation and carrying this extra load may also impact upon the energetic cost of locomotion. Here we demonstrate that winter birds have a reduced cost of locomotion when compared to summer birds. A remarkable finding given that during winter these birds have almost twice the body mass of those in summer. These results suggest that Svalbard ptarmigan are able to carry the additional winter fat without incurring any energetic cost. As energy conservation is paramount to these birds, minimising the costs of moving around when resources are limited would appear to be a key adaptation crucial for their survival in the barren Arctic environment.

## Introduction

Maintaining an optimal balance between energy acquisition and consumption is paramount to the evolutionary fitness of organisms [1], [2]. This is particularly pertinent in those species that inhabit areas of limited resources or that experience adverse environmental conditions, in which energy conservation becomes essential for survival. Being metabolically expensive, locomotion is a significant contributor to the daily energy budget of birds.

Locomotion in birds is complex as most species are capable of more than one mode of locomotion including flying, swimming, running and diving. Importantly, these different modes of locomotion are associated with morphological and physiological trade-offs [3], [4]. Research into avian locomotion has tended to focus on flight; however, many bird species spend the majority of their day either walking or running. In terrestrial locomotion, for an animal to move at greater speed (*U*) they must contract their muscles faster to move their limbs more quickly and reduce the amount of time that the feet are in contact with the ground [5], [6]. This requires more metabolic energy, which we can measure as oxygen uptake (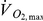). The amount of oxygen used (and therefore the metabolic rate) increases linearly with speed until the animal reaches its maximum rate of oxygen consumption 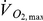 [7]–[14]. The efficiency of locomotion however, may vary in birds with differing locomotor specialisations, with both morphology and gait influencing the cost of locomotion [13]–[18]. Avian pelvic limb kinematics are broadly consistent across different species. For example, duty factor (DF) and the length of stance decrease with speed, whilst stride frequency and stride length increase, resulting in an overall decrease in contact time [14], [19]–[24]. More detailed analysis, however reveals important differences between species of differing size, posture [23] and locomotor specialisation [20], [25]. Smaller non-cursorial species, for example, show higher stride frequencies (*f*_stride_), shorter stride lengths (*l*_stride_) and higher DF for their size compared to larger, cursorial species and are more restricted in their speed range during different gaits [23]. Additionally, the number of gaits available to a species varies significantly. Some non-specialist birds, for example, are only able to walk across their entire speed range [15], whereas cursorial species are able to grounded run at intermediate speeds and aerial run at the top of their speed range [17], [19], [26]. Birds specialised towards non-terrestrial locomotor modes may use differing gait variations, such as the waddling walking gaits seen in penguins and mallards, characterised by extensive lateral movements of the centre of mass (CoM) [16], [20].

Some species of animal have been found to have an extraordinary capacity for load carriage [27]–[30]. Taylor et al., (1980) [31] tested rats, dogs, humans and horses and found that total metabolic rate increased in direct proportion to the added mass. However, upon conversion of this total metabolic rate to net metabolic rate (total metabolic rate minus the resting metabolic rate), the relationship becomes one of a fractional increase in metabolic rate that is greater than the fractional increase in mass [32]. Similar results have been observed in the small rodent, *Octodon degus* [33] and humans [34].

Artificial loading experiments in birds have shown that they are able to carry loads more efficiently than mammals, with either a less than directly proportional relationship [35] or a direct proportionality [32] between mass and net metabolic rate. Currently, data on avian load carrying are limited (only 2 species) [32], [35], [36] meaning further research is required to fully understand the underlying mechanisms.

The efficiency of load carriage is of particular relevance to avian species, many of which undergo seasonal or daily variations in mass, often in the form of fat reserves [37]–[39]. Fat deposition may be categorized into three major strategies; 1) seasonal or daily fluctuations in reserves to suit changing conditions, 2) pre-migratory fattening [40], [41], or 3) for reproduction and development [42]. Larger fat reserves are possibly due to increased unpredictability of food resources or longer migration routes [37], [38]. They are found at a number of inter-peritoneal and subcutaneous locations, for example at the furcula in small birds, the abdomen and pectoral girdle in others [43], [44]. These fat reserves are likely to incur both locomotor costs due to the need to move an increased body mass during locomotion and potentially a respiratory cost, as a result of heavier body components (such as the sternum and trunk walls) which must be moved during lung ventilation [45]. Indeed, sternal loading experiments suggest that the energetic impact of increased sternal mass may be substantial [32].

The trade-off between the need to maintain maximal fat reserves and the cost of carrying those reserves is likely to have large impacts upon the daily energy budget of birds. Any adaptations that minimise the cost of carrying these additional loads are likely to be evolutionarily advantageous [37]. Whilst a large body of work exists on the impacts of avian fat stores upon take-off and flight [46]–[48], no study to date has looked at the impacts of natural increases in mass upon terrestrial locomotor performance. Although the few artificial loading experiments performed on terrestrial birds may give some insight into the costs associated with increased fat mass, they do not represent the natural situation for fattened birds. Although the metabolic cost of fat carriage (i.e. ‘natural loading’) during locomotion in humans appears to be the same as the cost of artificial load carriage [34], [49], it is unclear if the same applies to bird species.

The Svalbard rock ptarmigan (*Lagopus muta hyperborea*) provides a unique opportunity for investigation into the effects of natural mass loading on the metabolic cost of locomotion. Rock ptarmigan are ground dwelling gallinaceous birds of the phasianid sub-family tetraonidae. These non-migratory birds inhabit the arctic archipelago of Svalbard year round [50]. The environmental conditions on Svalbard are extreme, with periods of continuous light from April to August and periods of continuous darkness between mid November and February. During the winter, food availability is unpredictable due to periods of mid-winter rain that can freeze, reducing the availability of food. Furthermore, vegetation on Svalbard is also low in biomass [51], [52]. Svalbard ptarmigan are well adapted to these hostile conditions most strikingly by undergoing profound seasonal changes in fat deposition. The addition of fat stores in preparation for winter, can comprise up to 32% of body mass [52]. Seasonal fluctuations in body mass appear to be a key adaptation for life on Svalbard as they are also observed in other over-wintering residents [53], [54]. In Svalbard ptarmigan these fat stores serve as emergency rations in times of low food availability rather than as a long-term source of energy [52], [55]. Fat stores may also serve as additional thermal insulation, and winter-insulated birds have a mass-specific conductance 43% below that predicted by body mass (significantly lower than equivalent summer values) [56], [57]. Changing photoperiodic conditions on Svalbard drive the physiological changes that result in the acquisition of winter fat. Svalbard experiences periods of continuous light (from April until mid-August) and dark (from mid-November until February) [58]. It is the vernal increase in day length that triggers the weight gain, beginning in August (at the end of the period of continuous light) and peaking in November (at the onset of continuous dark), upon which body mass declines gradually through the winter until March [59]. The role of the photoperiod in directing such physiological changes is not fully understood. However, it has been suggested to act via seasonal changes in the levels of melatonin and its knock on effects to the endocrine systems (i.e., thyroid and growth hormone) that are known to affect metabolism and fat deposition [60]–[62]. Interestingly, fattening occurs during a period when feeding levels are declining, reaching one third of their summer levels and although body mass then drops from November until April, food intake is doubled during February and March [63]. The observed changes in body fat composition are therefore thought to be a result of changing activity and energy expenditure rather than feeding levels alone [63], [64]. Mass specific RMR is 20% below summer values during winter, similar to other over-wintering species on Svalbard [53], [65]. The voluntary fasting and decrease in activity seen in Svalbard ptarmigan in winter enables decreased energy expenditure when energy conservation is key to survival and has been termed ‘arctic resignation’ [66]. Although behavioural adaptations may allow some conservation of energy during winter foraging [67], physiological mechanisms by which winter Svalbard ptarmigan may reduce the constraints imposed by fat are yet to be determined. Such mechanisms seem likely bearing in mind the importance of energy saving to the survival of these birds.

Here the impact of seasonal changes in body mass upon the energetics and kinematics of terrestrial locomotion in the Svalbard ptarmigan, *L. muta hyperborea* was determined. This study is the first to quantify the effects of ‘natural loading’ upon terrestrial locomotion in any animal other than humans [49], [68], [69]. We hypothesised that the ptarmigan will possess adaptations toward efficient load carriage in order to minimise the cost of locomotion during winter, when energy conservation may be critical for survival. Furthermore these adaptations should manifest as a lower metabolic cost of locomotion than expected given the increased body mass in winter birds.

## Materials and Methods

### Ethics Statement

All experimental procedures were covered by a UK Home Office project licence (40/3001) held by Dr Codd and under ethical approval of the National Animal Research Authority of Norway (permit number 1333/2008) and the University of Manchester.

### Animals

Captive adult male Svalbard rock ptarmigan (*L. muta hyperborea*) housed at the Department of Arctic Biology, University of Tromsø, Norway, were used for all experiments. Experiments were conducted on the same birds in summer (July 2009, n = 6) and winter (November 2009, n = 7). Svalbard ptarmigan were maintained indoors with *ad libitum* access to high quality food and water in line with previous studies [72], [73]. Artificial light and temperature conditions matched those in Tromsø, (69°46′N), with continuous light between May and August and temperatures within the thermoneutral zones of summer and winter birds, ensuring that they underwent their natural seasonal physiological changes [70]. The birds were not fasted prior to having the metabolic cost of their locomotion measured. Body mass was recorded throughout the experimental trials (summer: 491.97±10.42g; winter 721.5±16.38g, mean±SE). Prior to experiments, all birds were trained for at least 3 months to run upon a treadmill (Bremshey Trail Sport, Finland).

### Respirometry

O_2_ consumption (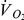) and CO_2_ production (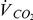) were measured using an open-flow through respirometry system [71], [72]. Ptarmigan were placed inside a Perspex® box (30×26×61.7cm) sitting upon a treadmill. Air was pulled through the box using a vacuum pump at a fixed flow rate of 52 l min^−1^. The main flow was then sub-sampled into a carboy at a flow rate of 6 l min^−1^ and then sub-sampled at 0.115 l min^−1^ for gas analysis. Relative humidity and water vapour pressure were recorded using an RH300 (Sable Systems International, Las Vegas, NV, USA). The air was then scrubbed of water using calcium chloride (2–6 mm granular, Merck, Darmstadt, Germany). CO_2_ measurements were then taken before the air was scrubbed of CO_2_ using SodaLime with indicator (2–5 mm granular, Sigma Aldrich, Steinheim, Germany) and finally O_2_ was measured. All gas analysis and data collection was performed using a FoxBox-C field gas analysis system (Sable systems International, Las Vegas USA). As water was scrubbed prior to gas analysis, the primary flow rate (FR) of the system was converted to a corrected flow rate (FR_c_) to account for the loss of water from the sample using eq. 1 (Eq. 8.6 in Lighton, 2008 [71]).
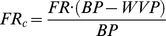
(1)Where *BP* is barometric pressure and *WVP* is water vapour pressure 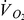 and 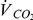 were calculated using eqs. 2 and 3 respectively [71].
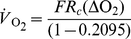
(2)

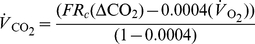
(3)Δ*O*_2_ is the difference between excurrent and background O_2_ concentrations. The respiratory quotients (RQ) of exercising birds were determined from these values as 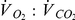 and used to calculate the rate of energy metabolism (W) [73]. These values were then divided by the mass of the bird to obtain the mass-specific metabolic power consumption during locomotion (*P*_met_, W kg^−1^).

Svalbard ptarmigan were exercised on the treadmill at increments up to the maximum speed attainable during both seasons (0.22–1.39 ms^−1^). Data were collected for 3–4 speeds on each day, with the speed and order of trial randomized and a rest day between trials. Birds were placed into the respirometry chamber and left to settle until a steady resting trace was obtained, defined by the oxygen consumption trace remaining at a steady plateau for at least 2 minutes. Data were then collected on each bird at a given speed until a stable measurement of gas concentrations was obtained, typically taking between 5–10 minutes. After each speed trial the bird was rested for 5–10 minutes until a stable resting trace was again obtained. The temperature of the room during trials was 18.58±0.18°C in the summer and 13.21±0.17°C in the winter, both values of which were within the birds' thermoneutral zones [57]. The accuracy of the respirometry set-up (±2% across all treadmill speeds) was validated using a N_2_ dilution test [74] as per our standard protocol [32].

### Kinematics

In order to obtain kinematic information, high-speed video footage was taken during all trials using a Sony Handycam HDR-SR8E (SONY, Japan) in summer and a Sony Handycam HDR-XR520 (SONY, Japan) in winter (at frame rates of 100 and 120 Hz respectively). Birds were filmed from a lateral view and the footfall events quantified using tracker.exe software version 2.6 (Open Source Physics) by tracking the left foot over 5–10 strides during which birds maintained a stable speed and position on the treadmill belt (i.e. they were neither accelerating nor decelerating). DF, *f*_stride_, *l*_stride_ and the length of the swing and stance phases (*l*_swing_, *l*_stance_ respectively) were the parameters calculated.

### Statistical Analyses

Statistical analyses were performed using the statistics toolbox in MATLAB®R2007b (The MathWorks Inc, Natick, MA, U.S.A.). Differences between seasons in both slope and intercept of the relationships between metabolic or kinematic variables and *U* were tested for using ANCOVA. When the slope was found not to differ between seasons, the ANCOVA was re-performed without the interaction term (season*U) i.e., assuming a common slope and testing for a difference in intercept only. All results are displayed as mean ± SE.

## Results

### Energetics

**Figure 1:**
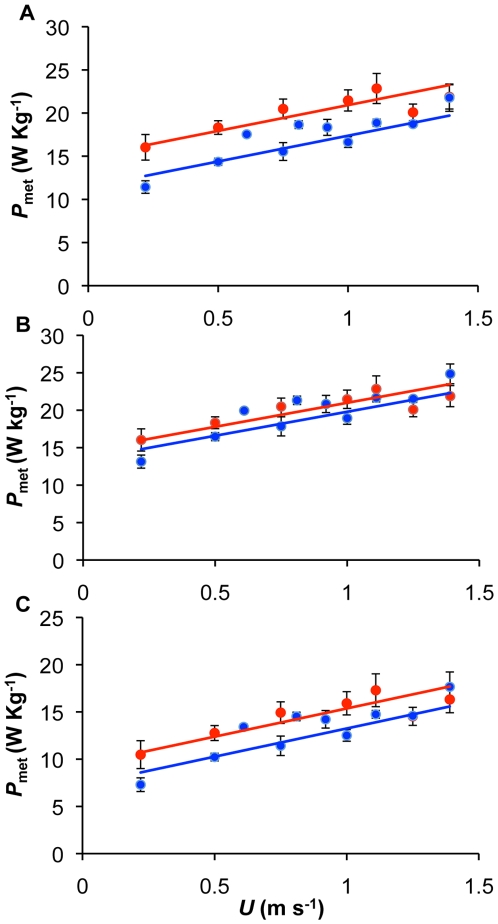
Mass-specific metabolic power consumption during locomotion (*P*_met_) plotted against walking speed (*U*) in winter (blue) and summer (red) acclimated birds. A) *P*_met_ increased linearly with *U* and was 31.05% higher in summer birds compared to winter birds, B) *P*_met_ values corrected for fat-free winter mass were not significantly different between the seasons, indicating a free cost of carrying the additional mass C) net *P*_met_ (calculated by subtracting resting metabolic rate values from *P*_met_ was significantly different between the seasons.

*P*_met_ increased linearly with running speed *U* (m s^−1^) between 0.22 and 1.39 m s^−1^ (Figure 1a) in both summer (*P*_met_ = 4.96*U*+15.81, t = 3.89, r^2^ = 0.32, p<0.01) and winter (*P*_met_ = 7.14*U*+10.57, t = 6.43, r^2^ = 0.57, p<0.001) birds. An ANCOVA showed no difference between the slopes of these lines, indicating that the incremental energetic response to increasing *U* was uniform between seasons (season*U, F_1,63_ = 1.62, p = 0.21). Accordingly, using the common slope and re-running the ANCOVA, showed the intercepts of the relationship between *P*_met_ and *U* were significantly different (ANCOVA: season, *F*_1,64_ = 29.03, r^2^ = 0.21, p<0.001; *U*, *F*_1,64_ = 48.65, r^2^ = 0.34, p<0.001), being 14.95 and 11.41 in summer and winter respectively. This represented a 31.05% higher *P*_met_ in summer compared to winter birds, despite winter birds being on average 47% heavier than those in the summer. In order to determine whether this increased mass was carried for free or not, winter *P*_met_ was corrected to W Kg^−1^ of fat free mass (Figure 1b) by subtracting an estimate of the dissectible fat present in winter birds [52] from measured values. Values for summer birds were not corrected as they have negligible fat levels. No significant difference was found between summer and winter birds indicating that they were carrying the additional fat at no additional energetic cost (Figure 1b, ANCOVA: season, *F*_1,64_ = 2.81, r^2^ = 0.025, p = 0.100; *U*, *F*_1,64_ = 47.7, r^2^ = 0.42, p<0.001).

### Correction for resting metabolic rate

In an attempt to account for the differences in *P*_met_ between summer and winter birds, net *P*_met_ was calculated by subtracting known mass-specific RMR values for summer and winter birds [57] from the experimental data. Again the intercepts of the regression lines of net *P*_met_ against *U* were significantly different, with values of 9.40 and 7.28 during summer and winter birds respectively (representing a 29.1% higher *P*_met_ in summer over winter values) (Figure 1c, ANCOVA: season, *F*_1,64_ = 10.38, r^2^ = 0.084, p<0.01; *U*, *F*_1,64_ = 48.67, r^2^ = 0.40, p<0.001). Thus differences in RMR do not account for the differing metabolic cost of locomotion in the seasonally adapted winter birds.

### Kinematics

**Figure 2:**
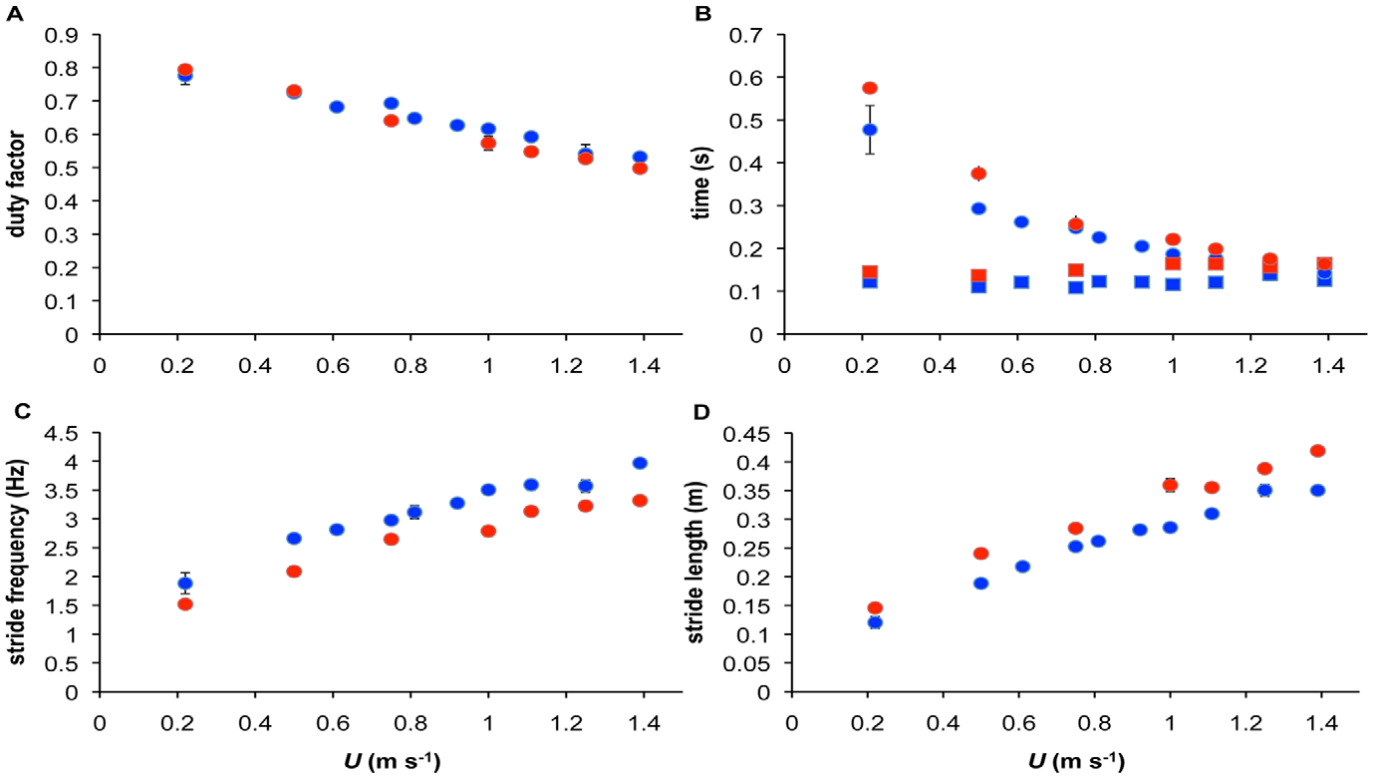
Gait kinematic parameters plotted against walking speed (*U*) in winter (blue) and summer (red) acclimated birds. A) Duty factor decreased with *U*, but never dropped below 0.5 across the speed range tested, B) The relative durations of the stance (*l*_stance_, circles) and swing (*l*_swing_, squares) phases. *l*_swing_ remained relatively unchanged across the speed range and was slightly reduced in winter birds. *l*_stance_ decreased curvi-linearly and was less in winter birds C) Stride frequency increased linearly with *U* and was 0.51Hz faster across the speed range during winter D) Stride length similarly increased linearly with *U* and was reduced in winter birds.

DF decreased linearly with *U* and the slope of the relationship was similar for both summer and winter birds (Figure 2a, ANCOVA: season*U, *F*_1,84_ = 7.36, r^2^ = 0.009, p = 0.008,). DF was, however, significantly lower during winter than summer (season, *F*_1,85_ = 6.08, r^2^ = 0.01, p = 0.0157; *U*, *F*_1,85_ = 663.01, r^2^ = 0.88, p<0.001), but never dropped below 0.5 over the range of *U* in either summer or winter birds, indicating an absence of aerial running. *l*_stance_ decreased curvi-linearly with *U* in both seasons (figure 2b) and was generally lower in the winter birds. The inverse of foot contact time (1/*t*_c_) increased linearly with *U* and the slope (3.87) of this relationship was the same for both winter and summer (ANCOVA: season*U, *F*_1,84_ = 0.4460, r^2^<0.01, p = 0.510). There was, however, a 0.61 s^−1^ decrease in 1/*t*_c_ in winter birds over the entire speed range (figure 2b, ANCOVA: season, *F*_1,85_ = 83.36, r^2^ = 0.04, p<0.001; *U*, *F*_1,85_ = 1740, r^2^ = 0.91, p<0.001).

*l*_swing_ increased slightly with *U* (figure 2b) and again the slope (0.015) of this relationship was common to both seasons (ANCOVA: season*U, *F*_1,84_ = 2.19, r^2^ = 0.01, p = 0.140,), with *l*_swing_ in winter birds being 0.03 s shorter across all speeds (figure 2b, ANCOVA: season, *F*_1,85_ = 117.7, r^2^ = 0.55, p<0.001; *U*, *F*_1,85_ = 11.78, r^2^ = 0.05, p = <0.001). *f*_stride_ increased linearly with a common slope of 1.62 in both seasons (figure 1c, ANCOVA: season*U, *F*_1,84_ = 0.39, r^2^<0.01, p = 0.540) and was 0.51Hz faster across the speed range in winter birds compared to those in summer (figure 2c, ANCOVA: season, *F*_1,85_ = 104.33, r^2^ = 0.14, p<0.001; *U*, *F*_1,85_ = 534.24, r^2^ = 0.74, p<0.001). Similarly, *l*_stride_ was positively correlated with *U* in both seasons with a common slope of 0.22 (figure 1d, ANCOVA: season*U, *F*_1,84_ = 0.99, p = 0.32, r^2^ = 0.00058), whilst being reduced by 0.04m in winter birds across the range of speeds (figure 2d, ANCOVA: season, *F*_1,85_ = 94.11, r^2^ = 0.06, p<0.001; *U*, *F*_1,85_ = 1530, r^2^ = 0.90, p<0.001,).

Hence, with the exception of DF, the incremental changes in kinematic parameters with speed did not differ significantly between seasons. The magnitude of these values across the range of *U*, however, did differ between winter and summer birds.

## Discussion

Svalbard rock ptarmigan undergo a dramatic seasonal change in body mass; the birds being up to 47% heavier during winter in the present study. Remarkably, carrying this extra load does not increase the *P*_met_. Indeed these birds are able to reduce the cost of locomotion in winter below that of summer birds. During winter the body composition of the Svalbard ptarmigan is around 30% fat [55]. Houston et al., (1997) [38] suggested that the observed patterns of fat storage in the Svalbard ptarmigan meant that either winter was not a relatively long time period for these birds or that the birds have a minimal or zero cost of fat storage. The results here present the first empirical evidence supporting this suggestion that carrying this extra fat load incurs no additional energetic cost during terrestrial locomotion in these birds. Mass-specific RMR during winter is 20% below summer values in the Svalbard ptarmigan [57], compared to 15% lower in arctic foxes [53] and 28% lower in Svalbard reindeer [75]. Such decreases in metabolic rate are less pronounced than in hibernating mammals [76]. Bouts of torpor have been reported in some bird species [77], [78], however it is currently unknown if Svalbard ptarmigan use this strategy.

Although decreasing RMR is a means by which animals are able to reduce energy expenditure, subtracting the differing inter-seasonal RMR values from *P*_met_ and calculating net *P*_met_ could not entirely account for the reduced cost of ‘naturally loaded’ winter birds: net *P*_met_ of summer birds was still 29% higher. This observed ‘free’ load carriage has not previously been reported in an avian species and has only been identified in a few other organisms [27], [79]. How free load carriage is achieved is not completely understood. However, one possible explanation lies in the patterns of exchange in the mechanical energies of the CoM during walking. Free carriage of loads up to 20% of body mass and exceptional efficiency in the carrying of larger loads upon the heads of women of the African Kikuyu and Luo tribes was suggested to be attributable to more effective pendular exchange between the kinetic energy of forward motion (*E*_kh_) and the sum of the gravitational potential (*E*_p_) and vertical kinetic energies (*E*_kv_) of the CoM during walking [30]. Similarly, energy savings during locomotion in the emperor penguin are brought about by efficient mechanical energy recovery during their waddling gait [16], due to increased lateral movements of the CoM. The possibility that improved pendular mechanisms and/or lateral movements of the CoM could contribute to the efficient locomotion of winter ptarmigan requires further investigation.

In addition to the potential for energy recovery via efficient pendular exchange mechanisms during walking gaits, are potential elastic savings during running gaits, in which *E*_kh_ and *E*_p_+*E*_kv_ are in phase and energy is recovered by storage and release in muscles and tendons [80]. Although changes in the efficiency of elastic energy savings during artificial loading are unknown, in unloaded animals elastic energy recovery can elicit large reductions in the cost of transport [81]. The possibility exists that the increased mass of the birds applies a greater force upon muscle and tendinous springs, eliciting a larger recovery of energy (in accordance with Hooke's law). Indeed, the physical properties of tendons themselves may alter in winter to facilitate improved elastic energy recovery. For example, increasing the mineralization of the tendons could facilitate improved elastic energy storage and load bearing capacity [82], [83]. Despite such possibilities, elastic energy storage seems unlikely as a means by which winter birds minimize the cost of running. The speed range covered in the present study encompassed the point of transition to grounded running in these birds (0.75–1.0 ms^−1^, JJL Pers. Obs July 2009), upon which we would expect the onset of elastic energy recovery and a resultant decrease in the slope of the regression line relating *P*_met_ to *U*. This decrease, however, was not observed with *P*_met_ increasing linearly and steadily over the range of speeds (and gaits) tested (Figure 1, a, b and c).

Seasonal modifications could also occur in the muscles. Contraction of the muscles involved in stance is the most significant contributor to locomotor cost [5]. Differing muscle types have different performance capacities. There is much plasticity in the muscular system and potential for translation from one fiber type to the other [84]. It is possible therefore that adaptations in muscles occur in winter Svalbard ptarmigan in order to compensate for increased body mass, however this remains to be determined. The present findings could be explained by an increase in the percentage of slow oxidative fibers during winter, which would increase the capacity for efficient locomotion at low speed but may restrict the top speed at which birds are able to locomote, due to an inability to generate the required locomotor forces swiftly enough. Indeed, winter Svalbard ptarmigan were unable to run with an aerial phase, as they were able to in summer.

Kinematic changes could also account for the lowered cost of locomotion in winter birds. As the cost of locomotion is inversely proportional to the time period available for the locomotor muscles to generate force [5], one would expect organisms of increased mass to alter their gait so as to lengthen the period of limb contact time with the ground. This would increase the time period available to generate force as a means of reducing the metabolic cost of load carriage. This is the case in artificially loaded horses, which increased contact time and stride period when loaded with 19% body mass [85] and in some species of birds and humans in which loading caused small but significant changes in *l*_stance_ and DF respectively [34], [35]. Other studies, however, have found differing results, indicating no changes in kinematic parameters upon loading [31], [32], [36]. The mixed results of these studies could be partly influenced by the artificial nature of their loading regimes, for example differences in load position and the stress associated with the addition of artificial loads. In the present study winter birds had significantly different gait kinematics from summer birds, exhibiting an increased *f*_stride_, shorter *l*_stride_ and reduced time of contact (Fig 1.). This seems counterintuitive as heavier birds are taking more frequent and shorter strides, decreasing the time available to generate muscular force during each step, which we would expect to be associated with an increased metabolic cost. The reasons underlying these kinematic changes are unknown but could be linked to non-energetic factors such as increased stability by minimizing the excursions of the feet from below the CoM, thereby decreasing stride length and creating a need for increased *f*_stride_ to maintain speed. Such stability may be of importance to these birds in the dark, icy winter environment on Svalbard. Alternatively, the shorter, faster strides in winter birds could be as a result of a change to a more cursorial, upright posture. Indian runner ducks have the same morphology as dabbling mallards (*Anas platyrhynchos*) yet are much more adept runners due to their upright posture and associated increased stride frequencies and decreased amplitude of locomotor movements [20]. The same kinematic changes were observed in the Svalbard ptarmigan potentially indicating a shift to a more upright posture, which could both serve to align the sternal mass with the CoM and also to improve mechanical advantage [86]. Winter Svalbard ptarmigan cannot run, even though their metabolic cost of locomotion was less, suggesting that there was not a metabolic constraint limiting top speed. Wild-type mallards and Indian runner ducks are able to run with an aerial phase [87]. However, the closely related but heavier Aylesbury duck (due to selective breeding for the meat industry) is unable to do so [87]. Aerial running is defined as a bouncing gait associated with an in phase relationship between the *E*_kh_ and *E*_p_+*E*_kv_, in which energy is stored and recovered via elastic elements [88]. It is possible that the increased weight of winter ptarmigan (and Aylesbury ducks) precludes aerial running as the forces generated during stance are too high to both be supported by the leg muscles and to allow realization of elastic savings. In this way, aerial running may overload the locomotor system, risking damage or even failure of the muscles and tendons.

Ambient temperature can have significant effects upon metabolism. For example a lowering of ambient temperature below the lower critical temperature is associated with an increase in metabolic heat production, typically through shivering thermogenesis because of the need to maintain heat balance [57], [89]. During locomotion, heat produced by working muscles substitutes heat production by shivering at low ambient temperatures, and under such conditions, locomotion may not be more energetically costly than staying at rest in a shivering state [90]–[92]. Conversely, running at high ambient temperatures may also incur an increased metabolic cost due to costs associated with the dissipation of excess heat (e.g., gular fluttering). Fowl, for example, experience a 20% increase in metabolic costs during terrestrial locomotion at temperatures of 32°C compared to a thermoneutral temperature of 20°C [93]. All birds in this present study were within their thermoneutral zones and it seems unlikely that the 5°C difference between summer and winter experimental temperatures is sufficient to explain the reduced *P*_met_ of winter birds. If anything, winter birds should be expected to display higher metabolic rates during locomotion than summer birds, due to their much better thermal insulation [60], and hence, larger need to dissipate excess heat through evaporative mechanisms. It is possible that the well-insulated winter birds may suffer from heat stress that in turn may restrict running at higher *U*.

Although the specific mechanisms underlying the apparent free cost of carrying large fat reserves in the Svalbard ptarmigan are unclear, its appears to be a key adaptation towards efficient locomotion which allows the conservation of energy when it is crucial and at its most limited. The fat stores of these birds are not sufficient to provide an energy source throughout the winter but serve as an emergency reserve and so they must still forage throughout this hostile season when food availability is unpredictable. By reducing the cost of locomotion, Svalbard ptarmigan are able to maintain this fat reserve for free and avoid an increased cost of foraging. The observed reduced cost of locomotion may help to explain the deposition of fat reserves themselves, as a small tip in the bird's energy balance toward storage rather than consumption in late autumn could aid in fat deposition, despite food intake being low [64]. Traditionally increased locomotor costs associated with the acquisition and maintenance of fat have been a factor in cost-benefit analyses of optimal fat reserves, despite there being no data regarding the terrestrial cost of fat storage [37], [38], [94], [95]. Although fat storage may have negative impacts upon flight performance and take-off [46], [47], the present findings suggest that birds may be able to limit its influence upon terrestrial locomotor performance. Many birds undergo seasonal fluctuations in body mass, some as extreme as those observed in the Svalbard Ptarmigan [42]. For example, some migratory birds may put on up to 40% of body mass as fat prior to migration [42]. The potential implications of the present study are therefore far reaching and more data is needed in order to see if our findings are a trend or an exception amongst birds.

In summary the Svalbard ptarmigan demonstrates exceptional natural load-carrying ability during winter, in which body mass is around 47% higher than during the summer, yet the mass specific cost of locomotion is 24% lower. Intriguingly the mechanisms underlying the efficiency of load carriage in these birds cannot solely be explained by the reduction in RMR that occurs during winter. It's possible that biomechanical factors may provide answers that go some way to helping explain this reduced cost. It would be interesting to determine whether other seasonally adapted birds are able to minimize the cost of locomotion in a similar way.
